# Relationship between polycystic ovary syndrome and high periostin level

**DOI:** 10.1590/1806-9282.20240138

**Published:** 2024-07-19

**Authors:** Semra Eroglu, Elcin Kal Cakmakliogullari

**Affiliations:** 1 Samsun University, Department of Gynecology and Obstetrics – Samsun, Turkey.; 2 Alanya Alaaddin Keykubat University, Department of Microbiology – Alanya, Turkey.

**Keywords:** Polycystic ovary syndrome, Periostin, Inflammation, Insulin resistance

## Abstract

**OBJECTIVE:**

There is growing evidence suggesting a relationship between periostin levels, inflammation, and ovarian dysfunction. In this prospective case-control study, we aimed to investigate serum periostin levels and their relationship with metabolic parameters in patients with polycystic ovary syndrome.

**METHODS:**

We conducted a prospective case-control study involving 45 polycystic ovary syndrome patients and 45 control subjects, matched in a 1:1 ratio. Serum samples collected from both study and control groups were analyzed using enzyme-linked immunosorbent assay.

**RESULTS:**

The demographic characteristics were similar between the polycystic ovary syndrome and control groups (p>0.05). Periostin levels were significantly higher in patients with polycystic ovary syndrome compared with the control group (4.67±2.46 vs. 2.60±1.41 ng/mL, respectively; p=0.000).

**CONCLUSION:**

Our study revealed a significant elevation in periostin levels among polycystic ovary syndrome patients compared with controls. These findings suggest that periostin could serve as a potential marker for assessing disease severity in polycystic ovary syndrome patients.

## INTRODUCTION

Polycystic ovary syndrome (PCOS) is the most common endocrinopathy in women during the reproductive period. Patients who meet at least two of the criteria of oligoanovulation, polycystic ovary appearance on ultrasound, and clinical or biochemical hyperandrogenism are diagnosed with PCOS^1^. Chronic inflammation is thought to play a role in pathophysiology. These patients suffer from insulin resistance (IR), obesity, dyslipidemia, infertility, and menstrual irregularities in their lives. The prevalence of obesity varies between 12.5 and 100% in publications^2^. In addition, there is an increase in the risk of type 2 diabetes, cardiovascular diseases, and metabolic syndrome (MS) with advancing age^3,4^.

Periostin is a matricellular protein that is embryonically expressed. It has been previously detected in bone, heart, teeth, uterus, and breast tissues^5^. The function of periostin is thought to be cell adhesion, migration, proliferation, and differentiation^6^. It is known that it survives longer than normal cells in hypoxic environments^7^. Periostin-secreting cells have been shown to increase paracrine tissue repair^8^. The relationship between PCOS, obesity, and chronic inflammation has been increasing in recent years^9,10^. It has been shown that periostin level increases with inflammation and IR^11^. It has been revealed that periostin may be involved in some metabolic diseases through JNK-mediated suppression of fatty acid oxidation in the liver^12^. Subsequently, periostin levels were shown to be strongly associated with triglycerides (TG) metabolism, chronic inflammation, and IR^13^. In addition, high glucose concentrations may also increase periostin expression. Besides this, studies on periostin in gynecology are increasing. Studies have shown that periostin increases in endometrial tissue in the midproliferative and early secretory phases and decreases in the late proliferative and late secretory phases. It has been suggested that periostin may play a role in implantation in the endometrium^14^.

In the literature, a few studies have examined the PCOS and periostin relationship^11,15^. The aim of this study was to determine the serum periostin level in PCOS patients and examine its relationship with metabolic parameters.

## METHODS

This is a prospective case-control study. Women between the ages of 18 and 45 years who applied to Karabuk University Training and Research Hospital Gynecology Polyclinic between April 2017 and June 2017 were included in the study. In this study, the participants were designated as the PCOS group (n=45) or control group (n=45) on a 1:1 ratio. The study protocol was approved by the ethics committee of the Medical Faculty of Karabuk University.

Polycystic ovary syndrome was diagnosed according to the 2003 Rotterdam European Society for Human Reproduction and Embryology (ESHRE)/American Society for Reproductive Medicine (ASRM) PCOS Consensus Workshop Group diagnostic criteria^1^. According to the NIH recommendation, there are four PCOS phenotypes evaluated: type A (hyperandrogenism, chronic anovulation, and polycystic ovaries); B (hyperandrogenism and chronic anovulation without polycystic ovaries); C (hyperandrogenism and polycystic ovaries); and D (chronic anovulation and polycystic ovaries without hyperandrogenism)^5,16^. In this study, the PCOS group consisted of phenotype-A patients. Pelvic ultrasound and standard gynecological examinations were performed by the same physician. All participants gave written informed consent.

The control group consisted of healthy individuals with regular menstrual periods, normal ovaries on ultrasonographic evaluation, and normal hormonal status who were matched for age and body mass index (BMI). Among the exclusion criteria were pregnancy status, malignancy history, hypothyroidism, hyperprolactinemia, Cushing's syndrome, congenital adrenal hyperplasia, inflammatory disease history, androgen-secreting tumor, active infection, and receiving treatment for PCOS or acne, including oral contraceptives, antidiabetics, acne or eyes solutions, glucocorticoids, anti-obesity, and ovulation induction agents. Age, height, weight, BMI, and menstruation findings were collected and recorded for all of the subjects. The BMI was calculated by dividing the weight (kg) by the square of the height (kg/m^2^).

### Sample processing

Blood samples were retrieved from all of the participants on the second and third days of the menstrual cycle after 8–12 h of overnight fasting. For the laboratory parameters, the ADVIA 1800 system (Siemens Healthcare Diagnostics Inc., Tarrytown, NY, USA) was used to evaluate the fasting and non-fasting glucose, TG, total cholesterol, high-density lipoprotein (HDL), and low-density lipoprotein (LDL) levels. IR was determined using the homeostatic model assessment of insulin resistance (HOMA-IR) index using the following formula: a [Fasting insulin (μU/mL) × Fasting Glucose (mg/dL)/405]. The serum periostin levels were stored at −70°C and determined by enzyme-linked immunosorbent assay (ELISA) (Fine test, Wuhan, Hubei, China).

### Statistical analysis

The SPSS 22 program was used for data analysis (IBM, NY, USA). The minimum sample size was calculated as 45 per group based on α error of 0.05, power of 0.90, and effect size of 0.7^15^.

The Kolmogorov-Smirnov test was used to determine normal distribution of the variables. As the data followed a normal distribution, the Student's t-test was applied to compare the clinical and laboratory parameters. The Pearson correlation analysis was performed to investigate the relationship between the variables. p<0.05 was considered statistically significant.

## RESULTS

Demographic and laboratory parameters of the PCOS and control groups are shown in Table 1. The mean age and BMI were similar in the PCOS and control groups (p=0.52 and p=0.864). Serum glucose levels and LDL cholesterol levels were observed to be significantly higher in the PCOS group compared with the control group (p=0.042 and p=0.03, respectively).

**Table 1:** Comparison between patients with polycystic ovary syndrome and the control group according to laboratory parameters.

Variables	PCOS group (n=45) Mean±standard deviation	Control group (n=45) Mean±standard deviation	p-values
Age (years, mean±SD)	22.40±5.46	23.02±4.30	0.52
Periostin (ng/mL)	4.67±2.46	2.60±1.41	0.000
Body mass index (kg/m^2^)	24.10±9.28	23.65±10.2	0.864
Insulin (mmol/L)	19.26±9.48	17.24±8.64	0.074
Serum glucose (mmol/L)	88.92±5.64	84.26±9.26	0.042
HOMA-IR	4.15±3.26	3.85±3.84	0.146
Serum cholesterol (mg/dL)	164.26±31.26	156.84±42.84	0.120
LDL cholesterol (mg/dL)	102.04±21.34	93.40±24.26	0.03
HDL cholesterol (mg/dL)	62.22±24.28	63.44±26.42	0.74
Triglycerides (mg/dL)	110.24±8.94	102.26±11.24	0.06
FSH (IU/L)	7.46±4.42	8.52±3.24	0.89
LH (IU/L)	9.52±5.28	8.62±4.42	0.06
Estradiol (pg/mL)	58.46±34.28	55.24±36.27	0.82
Total testosterone (ng/mL)	49.26±21.44	44.35±28.24	0.76

The parametric variables are shown as mean±SD, median. p-values were obtained using the Student's t-test. p<0.05 was considered statistically significant. HOMA-IR: homeostatic model assessment-insulin resistance; LDL cholesterol: low-density lipoprotein; HDL cholesterol: high-density lipoprotein; FSH: follicle-stimulating hormone; LH: luteinizing hormone.

The levels of periostin in the patients with PCOS and control groups were found as 4.67±2.46 and 2.60±1.41 ng/mL, respectively. The increased level of periostin in PCOS was also statistically significant (p=0.000).

There were no statistically significant correlations between periostin and metabolic variables according to correlation tests (Table 2).

**Table 2:** Correlation between periostin level and laboratory parameters in polycystic ovary syndrome patients.

	Periostin
Body mass index (kg/m^2^)	r=0.046 p=0.820
HOMA-IR	r=0.124 p=0.08
Serum cholesterol (mg/dL)	r=0.042 p=0.644
LDL cholesterol (mg/dL)	r=0.08 p=0.920
HDL cholesterol (mg/dL)	r=-0.62 p=0.54
Triglycerides (mg/dL)	r=0.47 p=0.26
FSH (IU/L)	r=-0.152 p=0.132
LH (IU/L)	r=0.162 p=0.09

p<0.05 is statistically significant.

## DISCUSSION

In this study, serum periostin levels were found to be significantly higher in PCOS patients. Hyperandrogenism and IR, which are involved in the pathophysiology of PCOS, are thought to be accompanied by chronic low-grade inflammation and are responsible for the reproductive and metabolic dysfunction in PCOS^9^. While polycystic ovarian morphology occurs intrauterinely, IR is usually the postnatal activating factor. Increased serine phosphorylation in the insulin receptor and disruption of the insulin signaling pathway to the ovary were seen as the primary cause. IR causes hyperinsulinemia by increasing ovarian androgen secretion or LH secretion^9^. Findings support some degree of IR in many women with PCOS. Maffazioli and colleagues identified the prevalence of MS in women with PCOS as 27.4%, with hypertension present in 10.9%. The prevalence of MS among normal weight, overweight, and obese women with PCOS was found to be 17.6, 22.6, and 33.9%, respectively^3^. The prevalence of MS appears to have increased in normal-weight individuals with PCOS compared with the general population. In this context, it will not be sufficient to address only menstrual irregularities and infertility issues in PCOS patients encountered in clinics. There is a need for new modalities to prevent or delay the formation of MS. Current treatments are aimed at addressing symptoms rather than resolving the cause of PCOS. For example, although ovulation can occur with ovulatory agents in PCOS, the chances of pregnancy can still be low. This is presumed to be due to the different effects of genetic and morphological changes on endometrial receptivity^10^. Therefore, different markers affecting the endometrium may play a role in physiology. The complexity of PCOS still remains a mystery.

Polycystic ovary syndrome has been divided into four phenotypes based on clinical or biochemical hyperandrogenism, ovulatory dysfunction, and polycystic ovarian morphology^16^. Differences in treatment methods in long-term PCOS follow-up studies may reveal factors affecting the progression to MS. Baracat et al. emphasized that anovulation and hyperandrogenism are necessary for the diagnosis of PCOS and argued that treatments may differ according to PCOS phenotypes. For example, letrozole may not be suitable for Phenotype C, as indicated by OKS Phenotype D^10^. In a study on 310 PCOS patients followed for 6 years, Soarez et al. used metformin for those with IR, and combined oral contraceptive (COC) and antiandrogen drugs for those without MS or reproductive desire, for menstrual regulation and hirsutism treatment, comparing them with the control group. Significant increases in the visceral adiposity index (VAI), indicating type 2 diabetes mellitus (DM) and cardiovascular risk, were observed only in Phenotype A. Despite treatment, there was no decrease in the risk of type 2 DM and MS in Phenotype A. The frequency of MS was found to be unchanged in all types except Phenotype D^4^. Phenotype D is considered the mildest type, and we believe that lifestyle changes and appropriate treatment can prevent future morbidities.

In another study, Iwata et al. divided PCOS patients into three groups in their treatment scheme: COC, Metformin, and COC+Metformin. Ultimately, they found that IR improved only in PCOS patients given Metformin treatment alone. No improvement in IR was observed in those using Metformin+COC treatment. However, using COC alone showed an improvement in acne, Ferriman-Gallwey index, and menstrual cycle index and a decrease in testosterone and androstenedione levels^17^. After that, Medeiros et al. published an article about dysglycemia prediction in PCOS patients. They enrolled 648 PCOS and 330 control subjects. In non-PCOS women, low levels of thyroid-stimulating hormone (TSH) and high levels of testosterone predict estimated average glucose (EAG), whereas in PCOS Phenotype A, low HDL cholesterol (HDL-C) and high estradiol levels predict EAG. In Phenotype D, EAG is predicted with high HDL-C. Finally, anthropometric, hormonal, and lipid parameters did not provide much benefit in predicting dysglycemia. The authors emphasized the need to evaluate outcomes across different phenotypes of PCOS^18^.

Similar to our study, Chen et al. demonstrated that periostin levels are elevated in PCOS patients and that there is a positive correlation between periostin and HOMA-IR^11^. Previous research has suggested that insulin may stimulate the theca cells of the ovary to produce excess testosterone, leading to clinical symptoms of hyperandrogenism such as acne, hirsutism, and alopecia^19^. Therefore, it is plausible to speculate that increased periostin levels may contribute to the development of IR in women with PCOS and play a role in the pathogenesis and clinical manifestations of the syndrome. Elevated periostin levels and chronic inflammation may explain hyperandrogenism. In Chen et al's study, glucose and LDL levels were significantly higher in the PCOS group, while HOMA-IR levels were independently associated with periostin. Moreover, periostin was positively correlated with BMI, uric acid, and HOMA-IR^11^. However, in our study, no significant relationship was found between metabolic parameters and periostin levels. Similarly, Gonulalan et al. also found no correlation between periostin and metabolic parameters^15^. Our findings are consistent with the existing literature. Based on these results, we believe that periostin levels may contribute to the pathophysiology of PCOS through chronic inflammation.

Periostin is a protein secreted from fibroblasts, which are a component of the extracellular matrix. It is known to be secreted from the lung, breast, thyroid, placenta, ovary, skin, and periodontal ligaments^5^. Its role in promoting the occurrence of various diseases is to activate different signaling pathways by combining with integrins. Recent studies have shown that periostin may be associated with inflammation, IR, glucose, and lipid metabolism. In addition, increased periostin expression was observed in the liver of obese rodents and humans and was associated with hepatosteatosis and hypertriglycidemia. Periostin overexpression was linked to decreased expression of peroxisome proliferator-activated receptor α (PPARα) protein^13^. Fatty acid oxidation caused by dyslipidemia can promote TG expression in the liver, while excessive activation of JNK signaling leads to the development of fatty liver, obesity, and IR^20^. Lu et al. revealed that periostin may be involved in some metabolic diseases through JNK-mediated suppression of fatty acid oxidation in the liver^12^. Subsequently, periostin levels were shown to be strongly associated with TG metabolism, chronic inflammation, and IR^13^. In addition, high glucose concentrations may also increase periostin expression. Glucose is the main redox substrate of cells in the mononuclear circulation. In this process, the formation of reactive oxygen species (ROS) is induced. This leads to the activation of NF-kB, the transcription factor that is associated with the expression of pro-inflammatory mediators such as TNF or IL-6^21^. Additionally, the hyperglycemic state may promote the secretion of steroidogenic molecules, which may result in hyperandrogenemia^22^.

It is believed that periostin plays an active role in the female reproductive system. Studies have shown that periostin is involved in various reproductive processes^14,23^. For instance, Hiroi et al. demonstrated in mice that periostin is crucial for implantation. Additionally, periostin mRNA levels in human endometrium fluctuate throughout the menstrual cycle, with significant increases during the mid-proliferative and early secretory phases and decreases during the late proliferative, mid-secretory, and late secretory phases. This suggests that periostin expression is regulated by ovarian steroid hormones in both rat uterus and human endometrium^23^. Furthermore, periostin mRNA levels have been monitored in the endometrium of pregnant sheep at 12–14 days, indicating a potential role for periostin in promoting trophoectoderm cell retention and migration, particularly in sheep treated with progesterone^24^. Subsequent studies have demonstrated that periostin enhances migration, adhesion, and invasion of endometrial tissues in the context of endometriosis^25^. In addition, periostin levels have been found to vary in cases of spontaneous abortion compared with voluntary abortions, suggesting a potential role for periostin in early pregnancy maintenance^6^. In our previous study, while serum periostin levels were lower in the spontaneous abortion group compared with the voluntary abortion group, tissue values were found to be similar^14^. Collectively, these findings indicate that periostin may act as a regulatory molecule in the female reproductive system, potentially influencing menstrual cycles, ovulation, and early pregnancy processes.

This study represents one of the initial investigations on periostin levels among patients with PCOS, thereby enriching our understanding of the interplay between PCOS pathophysiology, inflammation, and IR. Nevertheless, it is important to note that variations across different PCOS phenotypes were not explored in this study. In future studies, to comprehend whether elevated periostin leads to PCOS or if periostin levels elevate due to IR in PCOS, structural alterations that may occur can be monitored through electron microscopy or ovarian biopsy.

In conclusion, the levels of periostin are elevated in patients with PCOS. Periostin can serve as a marker for assessing the severity of the disease in PCOS patients. Understanding the role of periostin in the pathogenesis of the disease may pave the way for identifying therapeutic targets for future treatment of PCOS.
